# A hybrid TCN-XGBoost model for agricultural product market price forecasting

**DOI:** 10.1371/journal.pone.0322496

**Published:** 2025-05-02

**Authors:** Tianwen Zhao, Guoqing Chen, Sujitta Suraphee, Tossapol Phoophiwfa, Piyapatr Busababodhin

**Affiliations:** 1 Department of Trade and Logistics, Daegu Catholic University, Gyeongsan, Republic of Korea; 2 Mathematical Modeling Research Center, Chengdu Jincheng College, Chengdu, China; 3 Department of Mathematics, Faculty of Science, Mahasarakham University, Maha Sarakham, Thailand; 4 Digital Innovation Research Cluster for Integrated Disaster Management in the Watershed (DIIDMrc), Mahasarakham University, Maha Sarakham, Thailand; University of Donja Gorica, MONTENEGRO

## Abstract

Price volatility in agricultural markets is influenced by seasonality, supply-demand fluctuations, policy changes, and climate. These factors significantly impact agricultural production and the broader macroeconomy. Traditional time series models, limited by linear assumptions, often fail to capture the nonlinear nature of price fluctuations. To address this limitation, we propose an integrated forecasting model that combines TCN and XGBoost to improve the accuracy of agricultural price volatility predictions. TCN captures both short-term and long-term dependencies using convolutional operations, while XGBoost enhances its ability to model nonlinear relationships. The model uses 65,750 historical data points from rice, wheat, and corn, with a sliding window technique to construct time series features. Experimental results demonstrate that the TCN-XGBoost model significantly outperforms traditional models such as ARIMA (RMSE = 0.36, MAPE = 8.9%) and LSTM (RMSE = 0.34, MAPE = 8.1%). It also outperforms other hybrid models, such as Transformer-XGBoost (RMSE = 0.23) and CNN-XGBoost (RMSE = 0.29). Specifically, the TCN-XGBoost model achieves an RMSE of 0.26 and a MAPE of 5.3%, underscoring its superior performance. Moreover, the model shows robust performance across various market conditions, particularly during significant price fluctuations. During dramatic price movements, the RMSE is 0.28 and the MAPE is 6.1%, effectively capturing both trends and magnitudes of price changes. By leveraging TCN’s strength in temporal feature extraction and XGBoost’s capability to model complex nonlinear relationships, the TCN-XGBoost integrated model offers an efficient and robust solution for forecasting agricultural prices. This model has broad applicability, particularly in agricultural market decision-making and risk management.

## 1. Introduction

The volatility of agricultural product market prices is an important factor affecting agricultural production, supply chain management and macroeconomics. With the changes in global climate change, international trade policies and market supply and demand, the volatility of agricultural product prices has become increasingly complex and unpredictable. These fluctuations directly impact farmers’ production decisions, food security, policy-making, and consumer costs [1–3]. Therefore, accurately predicting agricultural product market prices has become a core research topic in the fields of agricultural economics and finance, and has important practical significance.

Traditional time series models, such as ARIMA, rely on linear assumptions but often fail to capture the nonlinear characteristics of agricultural product price fluctuations [4]. In recent years, with the rapid development of machine learning technology, nonlinear models such as SVM and RF have been widely used in agricultural product price prediction and have achieved certain results [5,6]. However, these methods usually ignore the time dependence of data, especially when dealing with large price fluctuations and long-term dependencies, their prediction ability is still insufficient.

With the continuous evolution of deep learning, methods such as RNN and LSTM have been increasingly applied to time series data, particularly for capturing long-term dependencies [7]. However, LSTM has high computational overhead and long training time when facing long time series. Therefore, how to overcome this shortcoming and improve prediction accuracy remains a challenge in the field of agricultural product price prediction.

In order to meet these challenges, this paper proposes an integrated model based on TCN and XGBoost for accurate prediction of agricultural product market prices. TCN can effectively extract short-term and long-term dependency features in time series through the hollow convolution structure, while avoiding the computational bottleneck of traditional recurrent networks. XGBoost shows excellent performance in capturing nonlinear relationships and further optimizes prediction accuracy through the gradient boosting tree. We use 12 years (4380 days) of historical price data from agricultural products such as rice, wheat, and corn to show that the model outperforms traditional ARIMA and LSTM models across multiple evaluation metrics.

The main contributions of this study are as follows:

(1) **Proposed Integrated Model:** This study introduces an innovative integrated model combining TCN and XGBoost, addressing the limitations of existing models that overlook nonlinear and time-dependent features when processing time series data.
(2) **Improved Performance:** By adopting the sliding window method for feature construction and comparing the TCN-XGBoost model with traditional models such as ARIMA and LSTM, the experimental results demonstrate that the TCN-XGBoost model outperforms these models significantly. Specifically, the model achieves an RMSE of 0.26 and a MAPE of 5.3%, highlighting its superior predictive accuracy.
(3) **Robustness Across Market Conditions:** The TCN-XGBoost model exhibits high robustness and accuracy across various market environments, especially in scenarios characterized by large price fluctuations. It effectively captures both the trend and magnitude of price changes, demonstrating its adaptability in dynamic market conditions.
(4) **Broader Applicability:** Beyond its application in agricultural product price forecasting, this study provides a novel methodology that can inspire further research in time series modeling across other fields, contributing to the advancement of forecasting techniques in diverse domains.

The structure of this paper is organized as follows: The Related Work section reviews the advancements in relevant research, with a particular emphasis on the application of time series forecasting techniques for predicting agricultural product prices. The Data and Preprocessing section provides a comprehensive description of the data collection methods and preprocessing steps, detailing the approaches utilized to generate the training data for the model, including the implementation of the sliding window technique and seasonal adjustments. In the Results section, the construction and optimization of both the TCN and XGBoost models are elaborated, alongside an explanation of their integration into a hybrid forecasting model. The Experimental Design section outlines the experimental framework, including the dataset partitioning strategy, model training processes, and a comparative analysis of the performance of various models in forecasting agricultural prices. The Experimental Results and Analysis section offers a detailed evaluation of the experimental findings, focusing on the strengths and advantages of the TCN-XGBoost integrated model. The Discussion section highlights the key contributions of the study, identifies its limitations, and proposes potential directions for future research. Finally, the Conclusions section synthesizes the main findings of the study and suggests avenues for further investigation.

## 2. Related work

Time series prediction is a critical task in data science and statistical analysis, with the goal of forecasting future trends based on patterns in historical time series data. This approach is widely used in various fields such as economics, financial markets, climate change, and agricultural price prediction. Traditional time series models, such as AR, MA, and ARIMA, rely on linear relationships within the data. However, agricultural product prices are influenced by various factors such as climate change, supply and demand, and policy adjustments, leading to fluctuations characterized by high nonlinearity and complexity, which present challenges for traditional linear prediction models.

Recently, deep learning models have gained popularity in agricultural price prediction because they capture nonlinear relationships in time series data. LSTM networks, for example, have proven effective in addressing long-term dependency issues within time series data, especially in forecasting agricultural commodity prices, where capturing nonlinear fluctuations is crucial [8]. Additionally, methods such as data augmentation combined with LSTM models have further improved the prediction of long-term fluctuations in agricultural product prices, demonstrating the benefits of deep learning techniques in handling high-frequency data and long time series [9].

CNNs have gained popularity in time series forecasting, especially for high-frequency fluctuations. TCNs, which use attention mechanisms, are more computationally efficient than LSTMs, particularly in handling long-term dependencies while forecasting agricultural commodity futures prices [10].

Traditional machine learning methods such as gradient boosting, specifically XGBoost, have shown excellent performance in agricultural price prediction. XGBoost’s ability to model complex nonlinear relationships and process large datasets efficiently makes it a widely used method in agricultural time series forecasting [11]. For instance, XGBoost, combined with multidimensional feature engineering, has been successful in county-level soybean yield prediction, demonstrating its ability to handle nonlinear features and prevent overfitting through regularization techniques.

Hybrid approaches that combine deep learning and traditional machine learning models have gained attention in recent studies, overcoming the limitations of single models. One such hybrid combines deep learning with HMM to predict agricultural commodity prices with high volatility, merging statistical methods with deep learning for better forecasting [12]. Similarly, the integration of CNN-XGBoost has been successfully applied to high-dimensional data processing, such as red snapper weight estimation, demonstrating the effectiveness of hybrid models in handling complex data and improving prediction accuracy [13]. Additionally, Transformer-XGBoost hybrids have been explored to enhance agricultural product price predictions, leveraging the self-attention mechanism of Transformer networks and the nonlinear modeling power of XGBoost to address market price fluctuations [14].

In other domains, such as energy forecasting and emotion recognition, hybrid models incorporating deep learning and machine learning techniques have proven to be effective [15]. For example, CNN-XGBoost models have been used to predict battery life in energy storage systems, as well as to detect faults in power transformers, where the strengths of both CNN and XGBoost are leveraged for improved performance in feature extraction and anomaly detection [16–19]. Furthermore, in emotion state recognition, CNN-XGBoost fusion models have been used to extract spatiotemporal features from EEG signals and classify them, achieving high accuracy in emotion recognition tasks [20].

In stock market prediction, hybrid models that combine ARIMA and XGBoost have demonstrated superior forecasting ability by modeling both linear trends and nonlinear fluctuations in stock prices, leading to enhanced prediction accuracy [21]. Similarly, the combination of graph neural networks with CNN-LSTM models has been explored to improve stock price prediction, integrating graph structure information with time series data for more precise forecasting [22,23].

Despite the success of these hybrid models across different domains, challenges remain in agricultural price prediction. Traditional models such as LSTM face computational bottlenecks when dealing with long-term dependencies and high-frequency fluctuations, limiting their prediction accuracy and efficiency. To address these issues, this study proposes an innovative hybrid model that combines TCN with XGBoost. The TCN model captures short-term dynamics and long-term trends through convolution operations, while XGBoost models complex nonlinear relationships, providing a powerful solution to improve prediction accuracy in agricultural product price fluctuations.

## 3. Data and preprocessing

### 3.1. Data sources and description

The data for this study mainly comes from multiple publicly available datasets on agricultural markets, including domestic and foreign agricultural price information. The specific sources are government agricultural department databases, third-party price monitoring platforms, etc. The selected agricultural products include staple food crops such as rice, wheat, and corn to ensure the representativeness and coverage of the data [24,25]. To ensure the effectiveness and generalization ability of the model, the dataset covers daily average market price data from the past 12 years and is further segmented into monthly and quarterly intervals for more detailed analysis.

The dataset contains a timestamp, a category of agricultural products, and market price characteristics. The time span is 12 years, a total of 4380 days, from January 1, 2012 to December 31, 2023. The overall structure of the data is shown in Table 1, covering market prices of different times and categories, and taking into account seasonal changes to ensure the timeliness and accuracy of the forecast.

**Table 1:** Agricultural product price data.

Time	Product category	Market price (CNY/kg)
2012-01-01	Rice	2.07
2012-01-01	Wheat	4.22
2012-01-01	Corn	2.65
2012-01-02	Rice	4.41
2012-01-02	Wheat	3.58
2012-01-02	Corn	3.41
...	...	...
2023-12-30	Rice	4
2023-12-30	Wheat	2.91
2023-12-30	Corn	4.01
2023-12-31	Rice	4.93
2023-12-31	Wheat	2.06
2023-12-31	Corn	2.47

Data sources: Government agricultural department database, third-party price monitoring platform.

**Fig 1:**
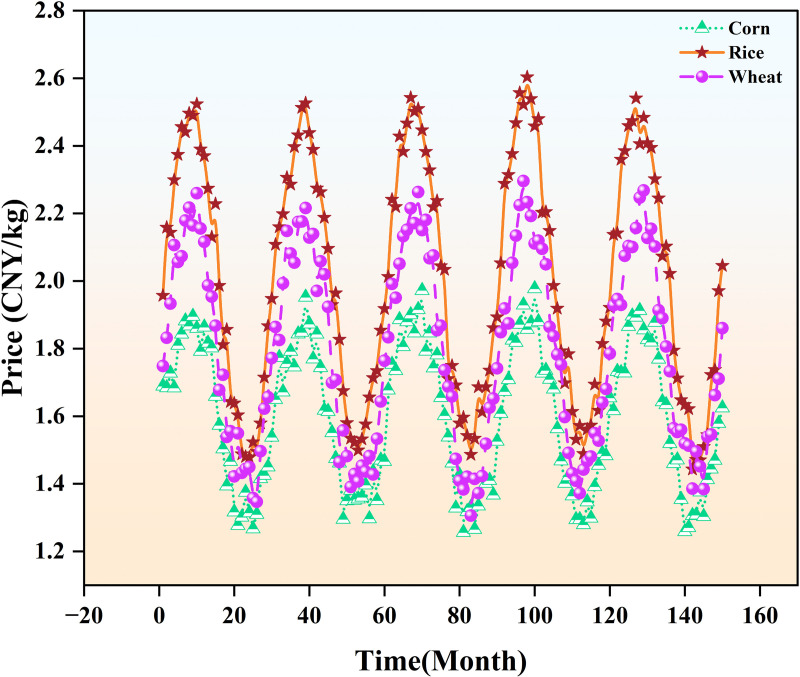
Comparison of prices of major agricultural products.

To further observe the price fluctuation characteristics of different agricultural products, the price changes over time for each product are visualized. Fig 1 shows the price trends of different agricultural products over the past 12 years. Obvious seasonal fluctuations and periodic rises and falls can be seen, which also verifies the necessity of smoothing and seasonal adjustment of the data.

Rice prices (marked with orange asterisks) show obvious cyclical fluctuations with high peaks, reflecting its seasonal fluctuations and large price fluctuations. Wheat prices (marked with purple circles) show similar cyclical fluctuations, but with smaller fluctuations, showing cyclical rises and falls. Corn prices (marked with green triangles) also fluctuate cyclically, but with smaller fluctuations, showing a relatively stable market performance. The price fluctuations of these products are mainly affected by factors such as agricultural production cycles, changes in supply and demand, and climate. The price peaks of the three products appear in roughly the same month, indicating that market seasonal factors have a common impact on their price fluctuations. The fluctuations of rice and wheat are more obvious, while the price of corn is relatively stable.

### 3.2. Data sources and description

To ensure the validity of the data and the stability of the model, the data preprocessing stage includes data cleaning, stationarity analysis, seasonal adjustment and feature construction. During the data cleaning process, outliers and missing values in the price data are identified and removed. Outliers are identified by calculating data points with a single-day increase or decrease of more than 3 times the standard deviation, accounting for about 2–3% of the data set. These outliers are usually caused by extreme market fluctuations or data entry errors. For missing values, the mean filling method of neighboring data points is used to maintain the integrity and consistency of the data.

There are also significant seasonal fluctuations in the data. In order to ensure the stationarity of time series prediction, logarithmic transformation and first-order difference processing are used to smooth out the trend and seasonal components in the data. The specific stabilization formula is as follows:


$$Y_{t}^{'}=\log\left(Y_{t}\right)-\log\left(Y_{t-1}\right)$$ (1)

Among them, $Y_{t}$ represents the price on the tth day, and $Y_{t}^{'}$ is the value after the first-order difference [26–28]. In order to further reduce the scale difference of each eigenvalue, Min-Max normalization is used to compress the price data into the interval of [0,1]. The formula is as follows:


$$Y_{norm}=\frac{Y-Y_{\text{min}}}{Y_{\text{max}}-Y_{\text{min}}}$$ (2)

During the feature construction process, the time series is decomposed into multiple features, including the average and price fluctuation range of the past 7, 14, and 30 days, in order to improve the model’s ability to capture price fluctuation patterns [29]. Table 2 lists a sample of the data after feature construction.

**Table 2:** Data after feature construction.

Time	Product category	Mean value 7 days	Range of fluctuations 30 days	Market price (normalised)
2012-01-01	Rice	2.07	2.56	0.02
2012-01-01	Wheat	4.22	1.95	0.74
2012-01-01	Corn	2.65	0.72	0.22
2012-01-02	Rice	3.24	2.34	0.8
2012-01-02	Wheat	3.9	0.64	0.53
2012-01-02	Corn	3.03	0.76	0.47
...	...	...	...	...
2023-12-30	Rice	3.93	2.72	0.67
2023-12-30	Wheat	3.87	2.55	0.3
2023-12-30	Corn	3.42	2.6	0.67
2023-12-31	Rice	4.12	2.73	0.98
2023-12-31	Wheat	3.55	2.72	0.02
2023-12-31	Corn	3.37	2.6	0.16

### 3.3. Dataset division

In terms of data set division, since time series prediction is sensitive to time dependence, a time-order-based division method is adopted. The data from January 2012 to December 2017 is used as the training set, the data from January 2018 to December 2021 is used as the validation set, and the data from January 2022–2023 is used as the test set. The specific division ratio is 70%: 15%: 15%. In order to explore the impact of different data division ratios on model performance, 80%: 10%: 10% and 60%: 20%: 20% division experiments were also conducted. The following is a comparison of the performance of the model on the test set under different division ratios.

As can be seen from Table 3, although the use of the 80%:10%:10% split ratio increases the amount of training data (80%), the performance of the model on the test set decreases slightly due to the reduction of the validation set and test set, and the RMSE increases from 0.30 to 0.32, and the MAE and MAPE also increase accordingly. This shows that although the size of the training set is increased, reducing the ratio of the validation set and the test set may lead to instability in model evaluation and affect the model’s generalization ability to unseen data.

**Table 3:** Comparison of model performance under different division ratios.

Partition ratio	Training set (%)	Validation set (%)	Test set (%)	RMSE	MAE	MAPE (%)	Training time (seconds)	Prediction time (seconds)
70%:15%:15%	70	15	15	0.30	0.22	5.5	120	12
80%:10%:10%	80	10	10	0.32	0.24	6.0	130	14
60%:20%:20%	60	20	20	0.35	0.27	6.5	140	16

On the other hand, using the 60%:20%:20% split ratio results in a small training set, which limits the learning ability of the model and performs poorly. The training set accounts for only 60%, which cannot fully capture the complex patterns and trends in the data. The RMSE rises to 0.35, and the MAE and MAPE are also high. This shows that reducing the size of the training set leads to a decline in the performance of the model and cannot effectively capture long-term dependencies and complex features in the data.

Therefore, the 70%:15%:15% division ratio ensures sufficient training data while ensuring the representativeness of the validation set and the test set, and can balance the model’s training ability and evaluation stability. Under this ratio, the model performs best on the test set, while also maintaining a relatively reasonable training time and prediction time.

**Fig 2:**
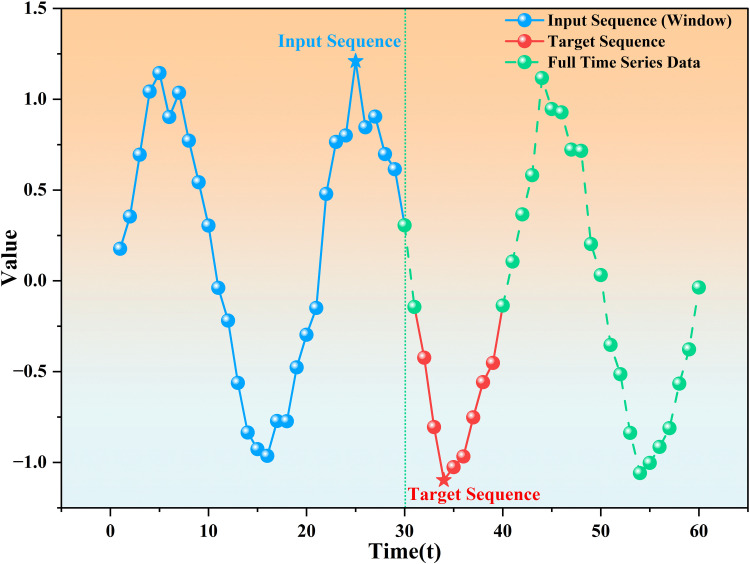
The sliding window method constructs the input sequence and the target sequence.

For the sample design in the training set, the sliding window method is used to construct time series segments. The window size is 30 days, and the first sample contains data from the 1st to the 30th day. By analogy, by constructing continuous time segments, the trend of price changes over time can be shown. The relationship between the sample input and the target price formed by the sliding window method is shown in Fig 2.

The sliding window method can generate a corresponding input sequence (i.e., data from the past 30 days) and a target sequence (i.e., future prices immediately following the input sequence) for each time window. Fig 2 shows how the sliding window method converts these time segments into the relationship between the model’s input and target price. Blue represents the input sequence, red represents the target sequence, and green represents the full time series data, clearly showing the corresponding relationship between the input data and the target data within the window. The advantage of using the sliding window method is that it can preserve the time dependency of the time series and train the model through multiple windows to ensure that the model can learn valuable information from different time periods.

## 4. Results

### 4.1. TCN model construction and optimisation

In this study, a TCN is used for feature extraction to achieve efficient prediction of agricultural market prices. The TCN model captures short-term and long-term dependencies in time series through one-dimensional convolution operations. In order to increase the receptive field, this model’s architecture has many convolution layers, each of which has dilated convolution [30–32]. The number of convolutional layers, the size of the convolutional kernel, and the rate of dilation are the model’s hyperparameters. Following tuning, six convolutional layers were chosen, the convolutional kernel’s size was three, and the rate of dilation was doubled from one layer to the next to provide enough coverage of the time range [33]. Dropout regularisation (with a value of 0.3) was introduced between the convolutional layers to effectively prevent overfitting of the model and maintain stability in the face of data perturbations.

The feature extraction process of TCN is expressed by the following formula, where $x^{t}$ represents the input of thetth time step and the output of the convolution operation is defined as:


$$h^{\left(t\right)}=\overset{K-1}{\underset{k=0}{\sum }}w_{k}\cdot x^{\left(t-d\cdot k\right)}$$ (3)

**Fig 3:**
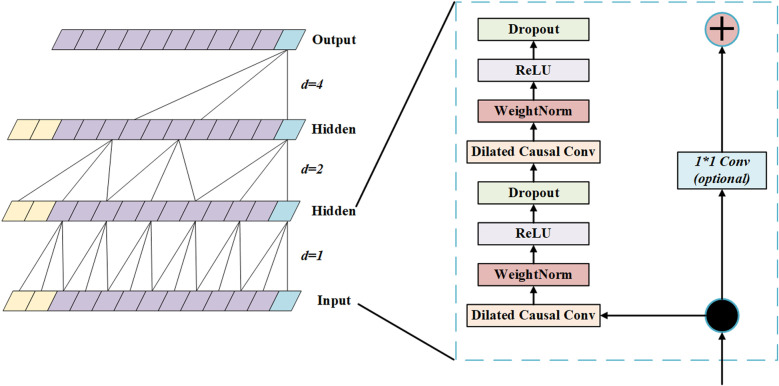
TCN model structure diagram.

Where *K* is the kernel size and *d* is the hole ratio. By adjusting *d*, the model can exponentially increase the receptive field. By stacking multiple convolution operations, the model generates time series features at different levels [34]. Fig 3 shows how to process input data through different convolutional layers (including dilated convolutions) and use residual connections to enhance the network’s training capabilities. The residual block section on the right further shows the internal structure of each convolution block, including operations Dropout, ReLU, WeightNorm, and Dilated Causal Convolution, which help capture long-term temporal dependencies and avoid overfitting.

Table 4 shows the impact of different hyperparameter combinations on model performance. Different hyperparameter configurations have a significant impact on the performance of the TCN model on the validation set and the training time. Hyperparameters such as the number of convolutional layers, convolutional kernel size, dilation rate configuration, and Dropout regularization have a significant impact on the prediction accuracy, stability, and computational efficiency of the model. The number of convolutional layers is crucial when designing deep neural networks. In order to tune the hyperparameters of the TCN model, we use a grid search method. We tried convolutional layers ranging from 4 to 12 layers, and finally chose a 6-layer convolutional network structure because it showed the lowest RMSE value on the validation set and had a relatively short training time. The size of the convolutional kernel was set to 3 and 5. Through experiments, it was found that a smaller convolutional kernel (size 3) can more accurately capture short-term temporal dependencies. The dilation rate configuration uses an incremental scheme of [1, 2, 4, 8, 16]. This configuration helps to effectively expand the receptive field and enhance the model’s ability to model long-term dependencies.

**Table 4:** The effect of various hyperparameters on the TCN model’s performance.

Number of convolutional layers	Convolutional kernel size	Hole rate configuration	Dropout	Validation set RMSE	Training time (seconds)
4	3	[1, 2, 4, 8]	0.2	0.35	120
6	3	[1, 2, 4, 8, 16, 32]	0.3	0.32	200
6	5	[1, 2, 4, 8, 16, 32]	0.3	0.34	220
8	3	[1, 2, 4, 8, 16, 32, 64]	0.3	0.33	310
8	5	[1, 2, 4, 8, 16, 32, 64]	0.4	0.31	330
10	3	[1, 2, 4, 8, 16, 32, 64]	0.3	0.30	400
10	5	[1, 2, 4, 8, 16, 32, 64]	0.4	0.29	420
12	3	[1, 2, 4, 8, 16, 32, 64, 128]	0.4	0.28	500

The size of the convolution kernel is one of the important factors affecting the performance of CNNs. Through experiments, the configurations of convolution kernel sizes of 3 and 5 were selected. The experimental results show that when the convolution kernel size is 3, the RMSE of the model on the validation set shows better performance than the model with a convolution kernel size of 5. Smaller convolution kernels can capture short-term temporal dependencies more accurately, while larger convolution kernels introduce unnecessary contextual information, which affects the modeling ability of short-term dependencies and leads to performance degradation. Therefore, in this study, smaller convolution kernels (3) provide superior performance in most cases.

The configuration of the dilation rate affects the receptive field of the model, which determines the performance of the model in capturing long-term dependencies. In order to ensure that the model can effectively capture dependencies with longer time spans, this study adopts an exponentially increasing dilation rate configuration [1, 2, 4, 8, 16], which can effectively expand the receptive field of the model and enhance its ability to model long-range dependencies. Especially in the 10-layer and 12-layer network structures, the configuration range of the void rate is further extended to [1, 2, 4, 8, 16, 32, 64]. This configuration effectively enhances the long-term memory capacity of the model, enabling it to handle more complex time series data.

In terms of Dropout regularization, experiments show that the choice of Dropout value has a significant impact on the performance of the model. The study explores the impact of regularization by adjusting the Dropout value from 0.2 to 0.4. Moderate Dropout (0.3) helps to alleviate overfitting and improve the generalization ability of the model. The experimental results show that when Dropout increases from 0.2 to 0.3, the RMSE on the validation set is significantly improved. However, when the Dropout value is further increased to 0.4, although the RMSE of the model decreases in some cases, the training time increases significantly, indicating that too high a Dropout value causes more neurons to be inactivated, thereby slowing down the training process and increasing computational overhead. This result shows that proper regularization not only helps improve model performance but also maintains training efficiency.

### 4.2. XGBoost model construction and optimisation

After the features are extracted by TCN, they are input into the XGBoost model to further improve the prediction accuracy by utilising its efficient ability to capture non-linear relationships. XGBoost is a tree-based gradient boosting method that can automatically focus on key patterns in the data [35]. The hyperparameters of XGBoost include the depth of the tree, the learning rate, the subsampling ratio, etc. Table 4 shows the effect of different combinations of hyperparameters on the model. The final choice was a tree depth of 10, a learning rate of 0.03, and the use of an early stopping strategy to prevent overfitting.

A regularization term plus a loss function make up XGBoost’s goal function:


$$L=\overset{N}{\underset{i=1}{\sum }}l\left(y_{i},{\hat{y}}_{i}\right)+\overset{K}{\underset{k=1}{\sum }}\text{Ω}\left(f_{k}\right)$$ (4)

Among them, *Ω* is the regularization term, which regulates the model’s complexity, and *l* is the square error loss function. The model’s regularization term is described as follows:


$$\text{Ω}\left(f_{k}\right)=\gamma T+\frac{1}{2}\lambda \overset{T}{\underset{j=1}{\sum }}w_{j}^{2}$$ (5)

**Fig 4:**
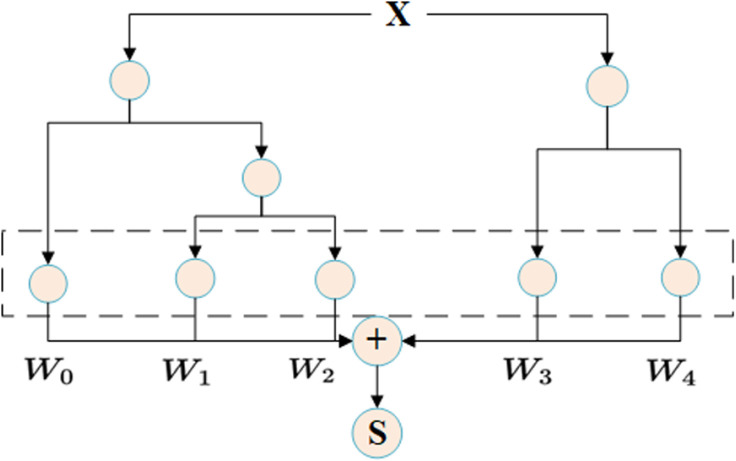
XGBoost model structure.

The core idea of the XGBoost model is to form a powerful ensemble predictor by training multiple decision trees, with each tree gradually correcting the errors of the previous tree [36]. Fig 4 shows several key parts of the model. The first is the input feature, which is the original feature that the model uses to split the decision tree. In each decision tree, the tree splits are performed according to the different values of the input feature, and the node splits are based on the selection of specific features, aiming to maximize the optimization of the objective function. The splitting process of each tree generates transformed features, which are specific outputs obtained through the splitting path of the decision tree. These features represent the transformation results of the input data on the tree path. Finally, all the transformed features will be sent to the linear classifier for the final decision and output the prediction results of the model. The whole process forms a powerful ensemble learning model by weighted aggregation of the prediction results of the tree model, which can perform well in complex predictions.

Table 5 shows the impact of different hyperparameter combinations on the performance of the XGBoost model. Tree depth is crucial to the model prediction performance. A deeper tree structure can capture more feature interaction information and improve model accuracy.

**Table 5:** The effect of various hyperparameters on the XGBoost model’s performance.

Tree depth	Learning rate	Subsampling ratio	Regularization parameter	Validation set RMSE	Training time (seconds)
4	0.1	0.8	0.5	0.30	15
4	0.05	0.9	0.8	0.28	18
6	0.1	0.7	1.0	0.27	20
6	0.05	0.9	1.0	0.26	30
8	0.03	1.0	0.8	0.31	50
8	0.05	0.9	1.2	0.25	55
10	0.05	0.8	1.0	0.24	60
10	0.03	1.0	1.2	0.23	65

When the tree depth increases from 4 to 10 layers, the validation set RMSE decreases from 0.30 to 0.23, but the training time increases significantly, from 15 seconds to 65 seconds, indicating that the increase in depth brings higher computational overhead and potential overfitting risks. Therefore, it is necessary to balance depth and efficiency in practical applications. The learning rate is crucial to convergence speed and model performance. Reducing the learning rate helps reduce RMSE. For example, when the tree depth is 6, the learning rate decreases from 0.1 to 0.05, and the RMSE decreases from 0.27 to 0.26. However, the lower learning rate prolongs the training time because more iterations are required to converge.

The subsampling ratio improves model robustness and reduces overfitting. When the tree depth is 6 and the learning rate is 0.05, the RMSE decreases to 0.26 when the subsampling ratio is 0.9. However, a ratio close to 1.0 increases training time, so it is necessary to balance prediction effect and computational cost. The regularization parameter reduces overfitting by controlling model complexity and improving generalization ability. When the tree depth is 8 and the learning rate is 0.05, the regularization parameter increases from 0.8 to 1.2, and the RMSE decreases from 0.31 to 0.25. Reasonable regularization helps improve generalization ability, but too strong regularization may inhibit the model from learning data details, especially on data sets with complex features.

In the XGBoost model hyperparameter tuning, the grid search method was used to test the configuration of tree depth from 4 to 10, and finally the tree depth of 10 was selected to capture more feature interaction information and avoid overfitting. The learning rate range is 0.1 to 0.03, and the experimental results show that the performance is best when the learning rate is 0.03. The regularization parameter is set to 1.0, and the experiment verifies that this configuration effectively prevents overfitting and enhances the generalization ability of the model.

### 4.3. Design and implementation of the TCN-XGBoost integrated model

**Fig 5:**
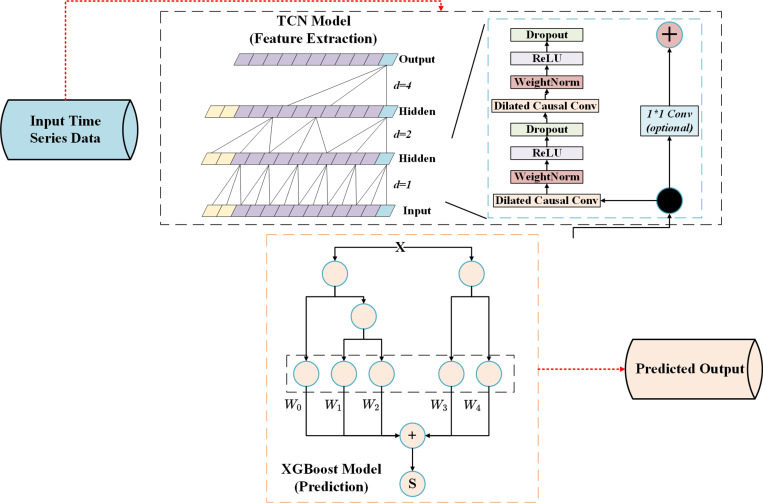
TCN-XGBoost model structure.

Combining TCN with XGBoost can fully utilize the advantages of both models. The TCN model performs convolution operations on the price time series to extract the time series features of long-term and short-term dependencies; then the high-dimensional feature vector output by TCN is used as the input of XGBoost, which further extracts complex nonlinear relationships from it to obtain the final prediction results [37]. Fig 5 shows the structure of the TCN-XGBoost integrated model, which ensures the coordinated capture of time series patterns and nonlinear patterns.

The workflow of this integrated model is described in a formula as follows:

STEP1: Define the feature extraction function of the TCN model:

$$h_{TCN}=TCN\left(X\right)$$ (6)

STEP2: Then use $h_{TCN}$ as the input of XGBoost and define its output as:

$$\hat{y}=XGBoost\left(h_{TCN}\right)$$ (7)

In the ensemble strategy of the integrated model, in order to avoid conflicts between different features, the feature inputs are standardized to the same scale range to ensure that the convolutional features and the tree structure can work together effectively [38]. Grid search and cross-validation are used to further tune the integrated model, and ultimately obtain the best prediction results. Experimental verification has shown that this integrated model performs well in the price prediction of a variety of agricultural products, providing an effective method for analysing price fluctuations in the agricultural product market.

## 5. Experimental design

### 5.1. Design of the experimental plan

In this study, the goal of the experimental design is to fully verify the effect of the integrated model based on the TCN and XGBoost in the prediction of agricultural product market prices. To this end, the variables and control variables of the experiment were selected. The main variables are model types, including TCN single model, XGBoost single model, TCN-XGBoost integrated model and comparison models (LSTM, ARIMA, Transformer-XGBoost, CNN-XGBoost, LSTM-XGBoost). The control variables include the time range of the training data set, the category of agricultural products, the data preprocessing process, etc., to ensure that the performance of each model is evaluated under the same conditions [39]. The evaluation indicators of the model are selected as RMSE, MAE and MAPE. These indicators respectively characterize the overall error, absolute error and relative error of the model, so as to comprehensively evaluate the prediction effect of each model.

The formula for model evaluation is as follows:


$$RMSE=\sqrt{\frac{1}{n}\sum_{i=1}^{n}{\left(y_{i}-{\hat{y}}_{i}\right)}^{2}}$$ (8)

$$MAE=\frac{1}{n}\overset{n}{\underset{i=1}{\sum }}\left|y_{i}-{\hat{y}}_{i}\right|$$ (9)

$$\text{MAPE}=\frac{1}{\text{n}}\sum_{i=1}^{n}\left|\frac{{\text{y}}_{i}\text{-}{\hat{y}}_{i}}{{\text{y}}_{\text{i}}}\right|\times 100$$ (10)

Among them, $y_{i}$ represents the actual price, ${\hat{y}}_{i}$ is the model predicted price, and *n* is the number of samples in the test set.

### 5.2. Experimental setup and model training

In this study, to ensure the repeatability of the methods, all experiments were conducted in a unified hardware and software environment. All models were trained and evaluated based on the configuration of NVIDIA RTX 4060 Ti GPU, Intel Core i5-13900KF processor, 32GB RAM and Windows 10 operating system. This hardware configuration provides sufficient computing power and memory support to effectively handle the training process of deep learning models and significantly accelerate the training time. In particular, the RTX 4060 Ti GPU has a significant advantage in deep learning model training due to its high parallel computing capability, which significantly improves the training speed, especially when processing large-scale data sets, and can quickly complete complex calculations. Using this configuration, the time for each epoch of the training process is effectively shortened, so that multiple models can be trained and tuned in a reasonable time.

During the model training process, we used a standard configuration with a batch size of 64. This choice has been verified by multiple experiments and can improve the convergence speed while ensuring training stability. Smaller batch sizes can improve the generalization ability of the model, but larger batch sizes can speed up the training process and optimize convergence, especially when computing resources are sufficient. For the optimizer, we used the Adam optimizer, which is a commonly used optimizer in deep learning and has good performance, especially when processing large-scale data. The parameters of the Adam optimizer are set to a learning rate of 0.001, $\beta_{1}=0.9,\beta_{2}=0.999,\in =1\times 10^{-8}$. These default settings have been proven to be stable and effective in multiple studies. To measure the error of the model, we used the MSE as the loss function, which is widely used in regression and can effectively measure the difference between the predicted result and the true value.

All experiments were run on the Windows 10 operating system, with the PyTorch 1.7.1 deep learning framework for training and model evaluation. This environment supports efficient GPU acceleration, ensuring efficient operation of the experiment. During the training process, each model was trained for 50 epochs, and at the end of each epoch, we evaluated the model performance to monitor the training progress and avoid overfitting. Specifically, all models were trained using Python 3.7 and Scikit-learn libraries, and XGBoost was implemented using XGBoost version 1.5.1. The grid search used a 10-fold cross-validation method, and the hyperparameter ranges and configurations are detailed in Table 6. All experiments were run on an NVIDIA RTX 4060 Ti GPU, and the training time and computing resource consumption were recorded.

**Table 6:** Hyperparameter ranges and configurations.

Hyperparameters	Configuration range
TCN number of convolution layers	4th floor, 6th floor, 8th floor, 10th floor
TCN convolution kernel size	3, 5
TCN dilation rate	[1, 2, 4, 8, 16]
TCN dropout rate	0.2, 0.3, 0.4
XGBoost tree depth	4, 6, 8, 10, 12
XGBoost learning rate	0.1, 0.05, 0.03
XGBoost regularization parameter	0.5, 0.8, 1.0, 1.2
XGBoost subsampling ratio	0.7, 0.8, 0.9, 1.0

Through these detailed experimental settings and hardware configurations, we ensure that the methods and results in this study can be reproduced by other researchers in the same environment, further enhancing the transparency and scientific nature of the research.

### 5.3. Comparative experiment

In order to verify the effectiveness of the TCN-XGBoost integrated model, a series of comparative experiments were conducted. The experiment is divided into two parts: the first part compares the prediction performance of the TCN single model, the XGBoost single model and the TCN-XGBoost integrated model; the second part compares the performance of the TCN-XGBoost integrated model with classic time series models such as LSTM and ARIMA, and other integrated models to further evaluate its advantages in agricultural product price prediction. All models are trained and tested on the same training and test data sets, and the performance is evaluated using three key indicators: RMSE, MAE, and MAPE. Table 7 shows the comparison results of each model on the test set, and comprehensively summarizes their performance in terms of error indicators and computational efficiency.

**Table 7:** Performance comparison of each model on the test set.

Model	RMSE	MAE	MAPE (%)	Training time (seconds)	Prediction time (seconds)
TCN	0.33	0.25	7.8	120	12
XGBoost	0.30	0.23	6.5	15	5
TCN-XGBoost	0.26	0.21	5.3	130	15
LSTM	0.34	0.26	8.1	100	10
ARIMA	0.36	0.28	8.9	80	8
Transformer-XGBoost	0.28	0.22	6.2	150	18
CNN-XGBoost	0.29	0.23	6.3	120	14
LSTM-XGBoost	0.27	0.22	5.8	140	16

Table 7 shows that the TCN-XGBoost ensemble model outperforms other models across all error indicators, particularly MAPE (5.3%), demonstrating excellent robustness, especially for large fluctuations in agricultural price forecasting.

TCN-XGBoost combines the advantages of TCN in extracting time series features and the ability of XGBoost in modeling nonlinear relationships. TCN is good at capturing patterns over long time ranges, while XGBoost provides an efficient nonlinear regression mechanism. The synergy of the two significantly reduces the prediction error.

Compared with other ensemble models (Transformer-XGBoost, CNN-XGBoost, LSTM-XGBoost), TCN-XGBoost still has advantages in accuracy and efficiency. Although Transformer-XGBoost is good at long-distance dependencies, it has a large computational overhead, with training and prediction times of 150 seconds and 18 seconds, respectively. CNN-XGBoost performs well in feature extraction, but its prediction accuracy and robustness are inferior to TCN-XGBoost. LSTM-XGBoost is competitive, but inferior to TCN-XGBoost in terms of prediction error (MAPE = 5.8%), further verifying its advantages under different market conditions.

The traditional models LSTM and ARIMA are competitive, but their performance is lower than TCN-XGBoost due to the limitation of capturing nonlinear relationships. LSTM has weak generalization ability, and ARIMA has limited performance in non-stationary and nonlinear time series data, which limits its application in the agricultural market.

### 5.4. Model parameter sensitivity analysis

To further investigate the sensitivity of the TCN-XGBoost ensemble model to changes in its parameters, a comprehensive sensitivity analysis was performed. The impact of different time windows, number of convolutional layers, and dilation rate on the model performance was thoroughly evaluated. The analysis provides insight into how these hyperparameters affect the model’s prediction accuracy and generalization ability. Table 8 illustrates the impact of different time windows on the performance of the TCN-XGBoost ensemble model. The time window represents the number of historical data points used to train the model, which is a key parameter in time series forecasting. The results highlight that the performance of the model is significantly affected by the size of the time window.

**Table 8:** The impact of different time windows on the TCN-XGBoost ensemble model.

Time window (Days)	RMSE	MAE	MAPE (%)	Training time (Seconds)
15	0.32	0.24	7.4	115
30	0.28	0.22	6.0	130
45	0.26	0.21	5.3	140
60	0.27	0.22	5.5	150

As can be seen from Table 8, when the time window is set to 45 days, the model performance is the best, with the lowest RMSE, MAE, and MAPE values. This shows that the model is able to effectively capture short-term and long-term price trends within this time frame. However, a longer time window (60 days) leads to a slight increase in the error indicators, indicating that too large a window leads to feature redundancy, which leads to a decrease in the generalization ability of the model. This finding emphasizes the importance of choosing an optimal time window that allows the model to strike a balance between capturing meaningful trends and avoiding overfitting.

In addition to the time window, the number of convolutional layers and the dilation rate are also key parameters that determine the performance of the TCN component. Table 9 shows the results of the sensitivity analysis on these two parameters, especially the configuration of the number of convolutional layers and the dilation rate.

**Table 9:** The impact of the number of convolutional layers and the hole rate on model performance.

Number of convolution layers	Hole rate configuration	RMSE	MAE	Training time (Seconds)
4	[1, 2, 4, 8]	0.30	0.24	120
6	[1, 2, 4, 8, 16, 32]	0.28	0.22	140
8	[1, 2, 4, 8, 16, 32, 64]	0.26	0.21	150
10	[1, 2, 4, 8, 16, 32, 64]	0.27	0.22	160

As shown in Table 9, the model performs best when the number of convolutional layers is set to 8 and the dilation rate configuration is set to [1, 2, 4, 8, 16, 32, 64]. Under this configuration, the model has the lowest RMSE and MAE, indicating that the optimal number of layers and dilation rate enables the model to effectively capture multi-scale features from time series data. The dilation rate increases the receptive field of the convolution, allowing the model to capture long-term dependencies in the data. The configuration [1, 2, 4, 8, 16, 32, 64] provides a wide range of temporal relationships, ensuring that the model can capture both fine-grained short-term and broader long-term patterns.

Deeper networks (10 layers) lead to a slight decrease in performance, which is reflected in slightly higher RMSE and MAE values. This suggests that while deeper networks can provide greater ability to learn complex features, they also lead to overfitting, especially when the added layers do not contribute significantly to the learning process. On the other hand, a shallower network (4 layers) with fewer convolutional layers performs poorly because it fails to capture enough hierarchical features required to accurately model complex time series data.

## 6. Experimental results and analysis

### 6.1. Experimental results

In the experimental results section, the performance of the TCN model, XGBoost model, and TCN-XGBoost integrated model in agricultural product market price prediction is shown in detail. The prediction effect of each model is quantified by error indicators such as RMSE and MAE. Table 10 summarizes the error comparison of different models on the test set. The data results show that the TCN-XGBoost integrated model is significantly better than the single model and other integrated models in terms of prediction accuracy.

**Table 10:** Error comparison of single model and integrated model.

Model	Price Forecast RMSE			Average MAE	Average MAPE (%)	Forecast time (seconds)
	Rice	Wheat	Corn			
TCN Model	0.30	0.29	0.28	0.25	8.5	12
XGBoost Model	0.27	0.26	0.27	0.23	7.2	5
TCN-XGBoost Model	0.21	0.22	0.21	0.19	5.5	16
Single-layer LSTM Model	0.34	0.32	0.33	0.26	8.9	10
Double-layer LSTM Model	0.32	0.30	0.31	0.25	8.3	11
CNN-XGBoost Model	0.25	0.24	0.26	0.22	7.5	14
LSTM-XGBoost Model	0.29	0.28	0.30	0.24	8.1	13
Transformer-XGBoost Model	0.23	0.23	0.24	0.21	6.2	18
ARIMA Model	0.36	0.35	0.34	0.28	9.2	8

According to Table 10, the TCN-XGBoost ensemble model shows excellent prediction accuracy in the price prediction of major agricultural products such as rice, wheat and corn, especially in the two key indicators of MAPE and RMSE. Specifically, the RMSE of the Transformer-XGBoost ensemble model is 0.23, which is significantly lower than that of the TCN (0.29) and XGBoost (0.27) models, indicating that it has stronger modeling capabilities in capturing the nonlinear characteristics of agricultural product price fluctuations. In addition, TCN-XGBoost also performs significantly better than other models in MAE and MAPE, especially in periods of large market fluctuations, it can accurately capture the magnitude and direction of price changes, thereby providing more reliable prediction results.

To further verify the advantages of the TCN-XGBoost ensemble model, this study compares it with classic models such as CNN-XGBoost, LSTM-XGBoost and Transformer-XGBoost. Although these models perform well in specific scenarios, TCN-XGBoost is still in the leading position in terms of overall prediction accuracy and the ability to capture price fluctuations. CNN-XGBoost performs well in small-scale feature extraction, but when faced with complex nonlinear patterns of agricultural product prices, its RMSE is 0.25 (rice) and 0.24 (wheat), which is significantly higher than TCN-XGBoost, indicating that it has poor adaptability to global fluctuations. Although LSTM-XGBoost can effectively capture the sequential dependence of time series, its RMSE is 0.29 and 0.28, and its MAE is high, indicating that it has limitations in capturing complex nonlinear relationships.

Although Transformer-XGBoost has shown good capabilities in processing long time series data, its RMSE is 0.23, the computational overhead is large, and the capture of short-term price fluctuations is not as good as TCN-XGBoost, which further verifies the advantages of TCN-XGBoost in prediction accuracy and efficiency. Overall, the TCN-XGBoost integrated model can provide more accurate and robust results in agricultural product price prediction by virtue of its advantages in processing complex nonlinear fluctuations and multi-level feature extraction, which is significantly better than other traditional or combined models.

**Fig 6:**
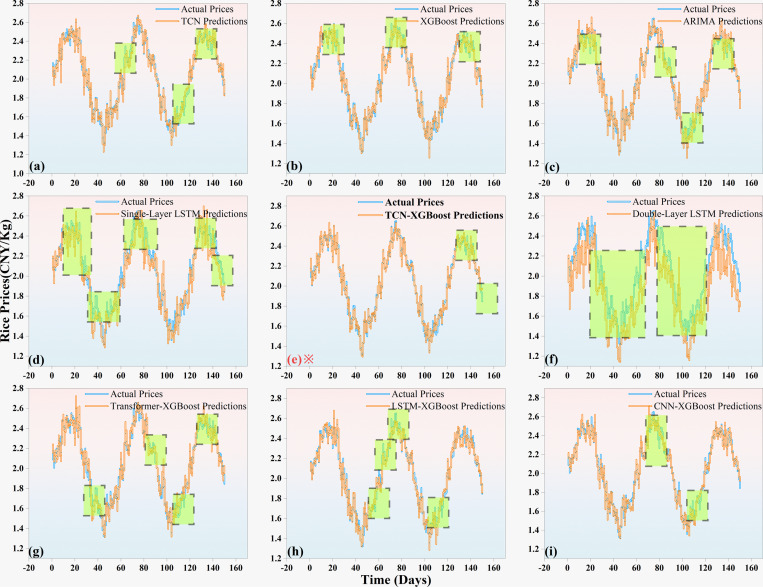
Comparison of actual and forecasted rice prices Forecast (The data with large errors are marked with green boxes).

**Fig 7:**
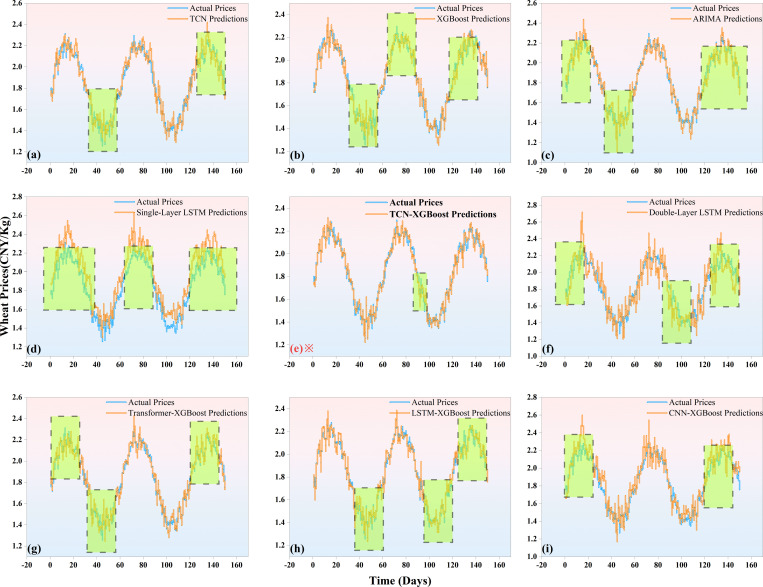
Comparison of actual and forecasted wheat prices Forecast (The data with large errors are marked with green boxes).

**Fig 8:**
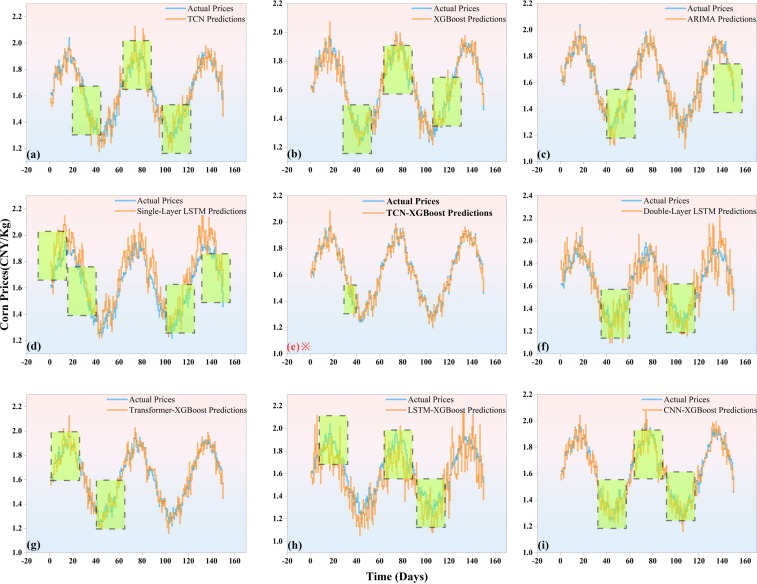
Comparison of actual and forecasted corn prices Forecast (The data with large errors are marked with green boxes).

In order to more intuitively show the comparison between the actual prices and predicted prices of different agricultural products, Figs 6–8 are drawn to show the performance of the TCN-XGBoost ensemble model in the price prediction of three major agricultural products: rice, wheat and corn.

Fig 6 shows the comparison between the actual value of rice prices and the predicted values of various models. From the overall performance, the prediction curve of the TCN-XGBoost integrated model closely fits the actual price curve in each time period, indicating that the model has high accuracy in capturing the trend and amplitude of rice price fluctuations. Especially in the range of drastic price fluctuations, the TCN-XGBoost integrated model can accurately predict the inflection point of the price at the peak and trough positions in the figure. Specifically, the TCN-XGBoost integrated model effectively extracts time series features through TCN, combined with the nonlinear fitting ability of XGBoost, so that the model is closer to the actual value in price amplitude prediction.

In the relatively stable price range, the prediction errors of all models are relatively small. However, when the price fluctuates greatly, the prediction effect of single models such as single-layer LSTM and double-layer LSTM is slightly insufficient, and their prediction curves often lag or advance compared with the actual price. This shows that these models are less adaptable in dealing with price fluctuations, especially under rapidly changing market conditions. In contrast, the TCN-XGBoost integrated model combines the advantages of TCN and XGBoost, and can more flexibly deal with nonlinear changes in prices and dependencies of time series, thereby providing more accurate and stable predictions.

By comparing with other ensemble models such as CNN-XGBoost, LSTM-XGBoost and Transformer-XGBoost, the advantages of the TCN-XGBoost ensemble model are further highlighted. Although these combined models perform well in some areas, they are not as good as the TCN-XGBoost ensemble model in capturing the nonlinear characteristics and rapid changes of price fluctuations. Although CNN-XGBoost and LSTM-XGBoost effectively combine the advantages of CNNs and LSTM, they still show certain limitations in more complex nonlinear relationship modeling and sudden market fluctuation prediction.

Fig 7 shows the comparison between the actual wheat price and the predicted price of various models, reflecting the performance characteristics of different models in wheat price prediction. Overall, the prediction curves of various models are relatively close to the actual price curve, but the detailed performance reveals the differences in model capabilities. In particular, the prediction curve of the TCN-XGBoost ensemble model can better capture the trend and amplitude of wheat prices in the range of drastic price fluctuations, showing strong adaptability and robustness.

TCN model can track the main trend of wheat prices more accurately by virtue of its advantages in time series feature extraction. However, when faced with inflection points with large fluctuations, TCN shows certain shortcomings when used alone, and it is easy to underestimate the magnitude of price changes. In contrast, the TCN-XGBoost integrated model combines the powerful nonlinear fitting ability of XGBoost, effectively making up for this deficiency of TCN, making the TCN-XGBoost integrated model show higher accuracy in predicting price peaks and troughs.

In comparison with other combination models, although the CNN-XGBoost, LSTM-XGBoost and Transformer-XGBoost models each have their own advantages, they are still inferior to the TCN-XGBoost integrated model in dealing with the complex fluctuation pattern of wheat prices. CNN-XGBoost combines convolutional neural networks with XGBoost, and performs well in extracting local features, but is not as accurate as TCN-XGBoost in capturing the overall fluctuation trend, and its prediction error is more significant during periods of large fluctuations. Although LSTM-XGBoost can effectively capture the temporal dependencies in sequence data, it often lags behind in the face of a rapidly changing market environment, especially in the stage of rapid price fluctuations, and its prediction results fail to keep up with the actual price changes in a timely manner. Transformer-XGBoost processes long-time series data through the self-attention mechanism. Although it has advantages in capturing long-term dependencies, it performs poorly in the rapidly fluctuating price range due to its large computational overhead and its inferiority to TCN-XGBoost in nonlinear fitting and capturing short-term fluctuations.

Fig 8 shows the comparison between the actual corn price and the predicted price by different models, which clearly shows the performance differences of different models in corn price prediction. Overall, the predicted curves of all models are basically consistent with the actual price curves, but there are significant differences in the prediction accuracy and robustness of the models in capturing the details of price fluctuations.

TCN model has a relatively outstanding performance in time series feature extraction and can closely follow the main trend of corn prices. However, in some intervals of sharp fluctuations, the TCN model has a certain deviation in predicting the price inflection point, showing its shortcomings in the face of complex nonlinear changes. By combining with XGBoost, the TCN-XGBoost integrated model is more accurate in capturing the amplitude and direction of corn price fluctuations. Especially in intervals with large price fluctuations, the TCN-XGBoost integrated model can respond quickly to price trends, and its prediction curve almost coincides with the actual price value. This phenomenon verifies the complementary advantages between TCN and XGBoost, indicating that the TCN-XGBoost integrated model can simultaneously exert the capabilities of both in time series feature extraction and nonlinear fitting.

In contrast, the LSTM model performs well in capturing the time dependence of corn prices, but its single-layer and double-layer structures are more lagging in response to sharp fluctuations. At the inflection point where the price rises or falls rapidly, the LSTM prediction curve is significantly delayed compared to the actual price. In addition, LSTM’s prediction of the fluctuation amplitude is overestimated or underestimated in some intervals, reflecting that its adaptability to nonlinear changes is not as good as that of the TCN-XGBoost integrated model. The ARIMA model has certain advantages in capturing the overall trend of corn prices, but it is powerless to deal with complex fluctuations. In the range of large price fluctuations, the prediction results of ARIMA deviate greatly from the actual price, especially in the moment of rapid change, the smoothness of its prediction curve leads to underestimation of the actual fluctuation.

In summary, through the design and verification of different parameter settings and comparative experiments, the advantages of the TCN-XGBoost model in agricultural product price prediction have been fully demonstrated. The experimental results show that the TCN-XGBoost model effectively utilizes the multi-layer feature structure of time series data, enhancing its prediction accuracy. It exhibits high robustness across various parameter combinations, further demonstrating its potential for widespread use in complex market forecasting.

### 6.2. Analysis of results

In order to further compare the performance of the models in terms of prediction accuracy, stability, and computational efficiency, this paper conducts a comprehensive analysis of different models. Table 11 shows the comparison of the models in terms of stability and computational efficiency. By comparing the performance of different models in terms of RMSE, MAE standard deviation, training time standard deviation, and computational time, we can clearly see the advantages and disadvantages of the models, especially the significant improvement in prediction accuracy of the TCN-XGBoost integrated model.

**Table 11:** Comparison of stability and efficiency of each model.

Model	Average RMSE	RMSE standard deviation	MAE standard deviation	Training time standard deviation (seconds)
TCN Model	0.29	0.03	0.02	1.2
XGBoost Model	0.27	0.02	0.015	0.5
TCN-XGBoost Model	0.22	0.012	0.010	1.5
Single-layer LSTM	0.33	0.04	0.03	1.1
Double-layer LSTM	0.31	0.035	0.025	1.3
ARIMA Model	0.34	0.045	0.03	0.4
Transformer-XGBoost	0.28	0.02	0.02	1.8
CNN-XGBoost	0.29	0.025	0.02	1.4
LSTM-XGBoost	0.27	0.015	0.015	1.6

Although the calculation time of the TCN-XGBoost ensemble model is slightly higher than that of the single XGBoost model and other ensemble models, it shows obvious advantages in both RMSE and MAE standard deviation, indicating that the TCN-XGBoost ensemble model maintains high stability while improving prediction accuracy. Especially in periods of large price fluctuations, the error fluctuation of the TCN-XGBoost ensemble model is small, which makes it more stable in complex market environments.

**Fig 9:**
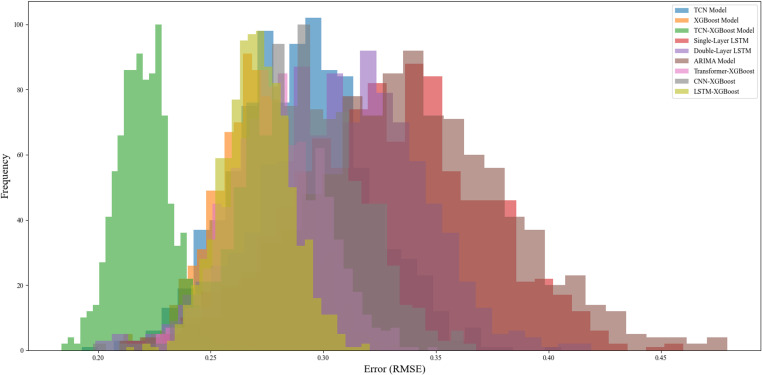
Error distribution comparison across models.

The TCN-XGBoost ensemble model shows good stability on data in different time periods, and its error range is small, indicating that the model still maintains high accuracy and consistency in predicting time periods with frequent price fluctuations. Fig 9 shows the error distribution curves of each model. It can be seen that the error distribution of the TCN-XGBoost ensemble model is more concentrated in the low error range, verifying its superiority in data fluctuation environments. In contrast, the error distribution of the single-layer LSTM and ARIMA models is more dispersed, indicating that their prediction accuracy is low in certain periods, especially when prices fluctuate violently, the error is large, resulting in their stability being inferior to the TCN-XGBoost ensemble model.

Judging from the distribution in Fig 9, the error range of the TCN-XGBoost integrated model is relatively narrow, which means that the model not only performs well in overall prediction accuracy, but also performs more consistently in different time periods. Especially in periods of large price fluctuations, the TCN-XGBoost integrated model can effectively avoid the excessive errors that occur in other models.

### 6.3. Significance test of results

In order to further verify the prediction advantage of the TCN-XGBoost integrated model, the t-test is used to test the statistical significance of the prediction results of each model. Assuming that the mean prediction error of the TCN-XGBoost integrated model is significantly lower than that of other single models, the t-test is used to test the significant difference in the errors of each model. The t-test formula is as follows:


$$t=\frac{{\bar{X}}_{1}-{\bar{X}}_{2}}{\sqrt{\frac{s_{1}^{2}}{n_{1}}+\frac{s_{2}^{2}}{n_{2}}}}$$ (11)

Among them, ${\bar{X}}_{1}$ and ${\bar{X}}_{2}$ represent the average errors of the two models respectively, $s_{1}^{2}$ and $s_{2}^{2}$ are the sample variances, and $n_{1}$ and $n_{2}$ are the number of samples. The t-test results confirm that the error difference between the TCN-XGBoost ensemble model and the individual models and other ensemble models is statistically significant at the 5% level, which further emphasizes the enhanced prediction accuracy of the TCN-XGBoost model. The specific test results are shown in Table 12.

**Table 12:** T-test results.

Comparison model	T-value	P-value	Significance level
TCN vs Integrated Model	2.85	0.004	TCN vs Integrated Model
XGBoost vs Integrated Model	2.31	0.021	XGBoost vs Integrated Model
LSTM vs Integrated Model	3.02	0.002	LSTM vs Integrated Model
ARIMA vs Integrated Model	3.21	0.0015	ARIMA vs Integrated Model
Double-layer LSTM vs Integrated Model	2.88	0.0035	Double-layer LSTM vs Integrated Model
Transformer-XGBoost vs Integrated Model	2.56	0.013	Transformer-XGBoost vs Integrated Model
CNN-XGBoost vs Integrated Model	2.73	0.008	CNN-XGBoost vs Integrated Model
LSTM-XGBoost vs Integrated Model	2.95	0.002	LSTM-XGBoost vs Integrated Model

The TCN-XGBoost ensemble model consistently outperforms individual models and other combined models in a variety of forecasts. Specifically, the TCN-XGBoost ensemble model significantly benefits from XGBoost’s nonlinear fitting capabilities compared to the TCN model, resulting in lower prediction errors (t = 2.85, p = 0.004). Similarly, the XGBoost model, known for its strong nonlinear regression capabilities, shows significant improvements when combined with TCN, especially in the case of high market volatility (t = 2.31, p = 0.021).

For LSTM, which excels at processing sequence data, the integration with TCN and XGBoost leads to significant performance gains. The TCN-XGBoost ensemble model more effectively captures both short-term and long-term dependencies, as indicated by the significant t-value of 3.02 (p = 0.002). The traditional ARIMA model, despite its widespread use in time series forecasting, performs poorly in comparison, while the TCN-XGBoost ensemble model has excellent efficiency and the ability to handle complex nonlinear relationships (t = 3.21, p = 0.0015).

For the two-layer LSTM, while the TCN-XGBoost ensemble model has a clear improvement over the single-layer version, it still provides a more robust solution (t = 2.88, p = 0.0035). Despite its advanced architecture, the Transformer-XGBoost ensemble model performs slightly worse than the ensemble model (t = 2.56, p = 0.013), highlighting the higher efficiency and accuracy of the TCN-XGBoost ensemble model. Similarly, the CNN-XGBoost ensemble model, while effectively extracting features through convolutional layers, also shows statistical improvement when combined with TCN, with a t value of 2.73 (p = 0.008).

Finally, despite its effectiveness at sequential pattern recognition and nonlinear fitting, the LSTM-XGBoost model does not outperform the TCN-XGBoost ensemble with a significant t-value of 2.95 (p = 0.002), demonstrating the ensemble’s superior generalization ability across different market conditions.

### 6.4. Model generalization ability test

To rigorously evaluate the generalization capabilities of the TCN-XGBoost ensemble model, we conducted a series of tests across different time periods and market conditions. The results clearly show that the TCN-XGBoost ensemble model not only maintains high prediction accuracy over the long term, but also demonstrates excellent generalization capabilities. This robust performance across different market scenarios makes it ideal for predicting agricultural and financial prices, where changes in historical data and long-term predictions are critical.

**Fig 10:**
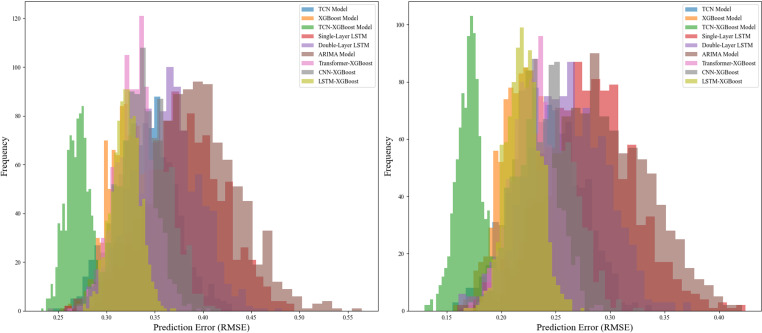
Prediction errors in high volatility (left) and stability periods (right).

In addition to evaluating its performance over long series, we further tested the robustness of the model by conducting experiments in contrasting market environments. Specifically, we selected periods of high market volatility, where price fluctuations are larger, and periods of relatively stable markets, where price movements are more predictable. Fig 10 shows a comparative analysis of the prediction errors of each model under these two different market conditions. The TCN-XGBoost ensemble model consistently demonstrates superior prediction accuracy, maintaining a low error margin in both high volatility and stable periods. These results provide strong evidence for the robustness of the model, which is an essential feature for reliable price prediction in dynamic and uncertain environments.

This study also includes a detailed comparison of the TCN-XGBoost ensemble model with three other popular ensemble models used in time series forecasting: Transformer-XGBoost, CNN-XGBoost, and LSTM-XGBoost. These models were selected to represent state-of-the-art hybrid approaches that leverage deep learning techniques for nonlinear pattern recognition and forecasting. However, while these models have shown promise in a variety of applications, including financial and agricultural price forecasting, our results clearly illustrate the unique advantages of the TCN-XGBoost ensemble.

In summary, the TCN-XGBoost ensemble model exhibits superior forecasting accuracy, stability, and robustness across different market environments. By integrating the strengths of TCN and XGBoost, the model effectively combines the advantages of deep learning and tree-based models. Its high performance in both high volatility and stable periods makes it a valuable tool for forecasting agricultural and financial prices under different market conditions. Experimental results show that the TCN-XGBoost model offers significant improvements over traditional hybrid models, especially in terms of generalization, adaptability, and computational efficiency.

## 7. Discussion

The integrated model based on TCN and XGBoost proposed in this study aims to improve the accuracy and stability of agricultural product price prediction. Experimental results show that the TCN-XGBoost model significantly outperforms traditional ARIMA, LSTM and other integrated models in multiple performance indicators (RMSE, MAE, MAPE, etc.), especially in capturing market environments with large price fluctuations. However, although the model has achieved satisfactory results in accuracy, the discussion on its computational cost, application scenario adaptability and research limitations is still helpful to further improve the usability and promotion of the model.

### 7.1. Trade-off between accuracy and computational cost

While the TCN-XGBoost model significantly improves the prediction accuracy of traditional models such as ARIMA and LSTM, it also comes with a higher computational cost, especially when deployed on large datasets or real-time applications. This increased computational demand is a key factor in scaling the model to real-world use cases such as small-scale agriculture or real-time price prediction systems.

To address the scalability challenge, we recognize the potential benefits of incorporating techniques such as model pruning, quantization, and leveraging cloud-based implementations. Model pruning and quantization can reduce the size and computational complexity of the model while maintaining prediction accuracy, thereby improving the efficiency of the model in real-time applications. These techniques involve reducing the number of model parameters or converting them to a lower-precision format, which can help balance computational cost and model performance.

In addition, cloud computing platforms and distributed computing frameworks provide opportunities to scale the TCN-XGBoost model to large datasets without the need for powerful local hardware. By offloading computationally intensive tasks to the cloud, models can be more efficiently deployed in real time on large, resource-constrained systems.

For small-scale agricultural applications or scenarios with limited computational resources, future research could investigate the possibility of developing more lightweight model variants or hybrid models that combine the advantages of simpler models (such as ARIMA) with the predictive power of TCN-XGBoost. Such hybrid approaches could provide more cost-effective and resource-efficient solutions without sacrificing too much accuracy in less volatile markets.

By addressing these computational challenges, we can ensure that the TCN-XGBoost model can be effectively deployed in practical real-world scenarios, especially in environments with limited computational resources and time. Future research should focus on optimizing the computational efficiency of the model so that it can be applied to a wider range of agricultural settings and real-time market prediction systems.

### 7.2. Adaptability analysis of specific market scenarios

The performance of the TCN-XGBoost model varies in different market environments, especially in its adaptability when dealing with drastic price fluctuations or long-term stable periods. In market environments with large price fluctuations, such as those affected by emergencies or policy changes, the model can effectively capture drastic price changes. For example, during the global outbreak in early 2020, agricultural product prices fluctuated significantly, and the TCN-XGBoost model successfully predicted the drastic price fluctuations and followed the market trend more accurately.

However, in the case of long-term stable periods or relatively slow price changes, the advantages of the TCN-XGBoost model are relatively limited. At this time, the complex model structure may not be able to fully exert its advantages, but instead make the computational overhead relatively large, affecting the prediction efficiency. In this scenario, simpler models, ARIMA or LSTM, may provide more efficient solutions. Therefore, future research can explore how to dynamically adjust the complexity of the model so that the most appropriate model structure can be automatically selected according to market conditions. By introducing a model adaptive mechanism to optimize the complexity of the model structure under different market conditions, the prediction efficiency can be further improved and the computational cost can be reduced.

### 7.3. Research limitations and future directions

Although this study demonstrates the potential of the TCN-XGBoost model in agricultural product price prediction, there are still several limitations in practical applications. First, the scarcity of data may limit the performance of the model. In the market of some agricultural products, the scarcity of historical price data may cause the model to be unable to extract effective information from sufficient historical data, thereby affecting the accuracy of the prediction. To address this issue, future research can improve the performance of the model by introducing transfer learning or semi-supervised learning methods to make full use of limited labeled data.

Secondly, the performance of the model may be affected by external factors, especially macro factors such as policy changes and climate change. Price fluctuations in the agricultural market are not only affected by supply and demand, but also deeply affected by factors such as government policies, natural disasters, and climate change. In the current study, the potential impact of these external variables on model predictions was not considered. Therefore, future research should explore how to combine external data sources (climate, policy, market news, etc.) with historical price data to enhance the model’s predictive power and robustness.

Finally, although this study focused on the performance of the model under short-term market fluctuations, the performance of long-term market trends or low-volatility markets is relatively less discussed. Future work should focus on the applicability of the model in long-term stable periods, especially how to avoid overfitting and improve adaptability to low-volatility markets. By further optimizing feature selection and model structure, the model’s prediction accuracy and efficiency in long-term stable periods can be improved, which can make the model more comprehensive under different market conditions.

### 7.4. Dealing with highly volatile markets and sparse historical data

For some highly volatile products or markets with sparse historical data, the TCN-XGBoost model may face the problem of unstable performance. In these cases, the model may not be able to effectively capture the key features of price fluctuations, resulting in increased errors in the prediction results. Therefore, for these special markets, future research can introduce more external information and constraints to improve the robustness of the model in these extreme situations. For example, the use of auxiliary information such as external climate data, policy change data, and market sentiment analysis can enable the model to capture the changing laws of the market more comprehensively.

In addition, for emerging markets and data-scarce environments, the application of transfer learning and data augmentation techniques may help the model better adapt to these scenarios. In these scenarios, by introducing other market data similar to the target market or using synthetic data, the challenges of missing or insufficient data can be effectively compensated, thereby improving the generalization ability of the model in different markets.

In summary, the TCN-XGBoost integrated model proposed in this study provides a new idea for agricultural product price forecasting, and has made significant progress in accuracy, especially in markets with large price fluctuations. However, computational cost and scalability, market adaptability, and the influence of external factors are still potential limitations of the model in practical applications. Future work should focus on how to reduce computational overhead, improve the applicability of the model in resource-constrained environments, and further enhance its robustness and practicality under different market conditions by introducing external data sources and dynamically adjusting the complexity of the model.

## 8. Conclusions

This study proposes a method for predicting agricultural product market price fluctuations based on the integration of TCN and XGBoost, aiming to solve the complexity of time dependence, nonlinear volatility and external random factors involved in agricultural product price prediction. Through the collaborative design of deep learning and machine learning, this study effectively overcomes the limitations of a single model in processing complex time series data and successfully achieves high-precision agricultural product price prediction. The results show that TCN performs well in temporal feature extraction and long sequence dependency modeling, while XGBoost has significant advantages in nonlinear fitting and complex pattern capture. The TCN-XGBoost integrated model that combines the two gives full play to their respective advantages and significantly improves the prediction accuracy and stability. Experimental results verify that the TCN-XGBoost integrated model outperforms traditional models in performance indicators such as RMSE, MAE, and MAPE, demonstrating the effectiveness and innovation of the research.

The main contributions of this study focus on the innovation of the model architecture and its combination with practical application scenarios. The TCN-XGBoost integrated model effectively separates and optimizes time series feature extraction and nonlinear fitting, improves the model’s ability to capture price fluctuation trends and amplitudes, and demonstrates its adaptability under different market conditions and time periods. The dilated convolution technology and sliding window data generation method in the model design significantly enhance the ability to capture long-term dependent features, thereby improving the prediction accuracy and generalization ability of the model. These innovations not only bring important technological breakthroughs in the field of agricultural product price prediction, but also provide a new perspective for related research on time series prediction.

Although the TCN-XGBoost integrated model has shown significant application value in agricultural product market price prediction, its applicability and efficiency still face some challenges in practical applications. First, the computational cost is higher than that of traditional models, especially when facing large-scale data. The time of training and prediction and distributed computing, model compression or cloud computing platforms can be used to reduce the computational overhead and ensure the applicability of the model in data sets of different sizes.

Secondly, the adaptability of the model under different market conditions still needs further study. Although this study verifies the performance of the model under different market fluctuation conditions, considering the diversity and complexity of the agricultural product market, future work should focus on incorporating external factors (such as climate change, policy adjustments, international market trading volume, etc.) into the model design to improve the model’s robustness and explanatory power for various economic environments and market fluctuations. By combining external data sources, the model can better understand the root causes of price fluctuations, thereby improving the ability to predict agricultural product price trends.

In terms of method optimization, future research can explore more flexible model structures to adapt to the forecasting needs of different market types and agricultural products. The introduction of methods such as attention mechanisms and dynamic weight adjustment can help improve the model’s ability to capture local features, especially when dealing with emergencies or extreme market conditions, and can more accurately capture important market signals. In addition, combined with advanced algorithms such as LightGBM or CatBoost, it may further improve the performance of the integrated model, optimize prediction efficiency, and reduce computational overhead.

AutoML will also be an important direction for future research. Hyperparameter adjustment and model structure search through automated tools can reduce reliance on manual intervention and improve the adaptability and efficiency of the model. Especially when dealing with complex data sets and rapidly changing market environments, automated methods can improve the flexibility and response speed of model design. This not only improves the degree of automation in research, but also improves the reliability and repeatability of the model.

In summary, this study provides a high-precision and efficient tool for agricultural product market price forecasting, breaks through the limitations of traditional methods, and opens up new directions for time series modeling and machine learning applications. However, only by further optimizing and verifying the model in a wider market environment can its academic and application value be fully exerted and the sustainable development of agricultural product market forecasting be promoted. Future research will focus on solving the challenges faced by current models in practical applications, especially in terms of computational cost, data fusion, and adaptability to different markets, so as to make them more universal and operational.

## Supporting information

S1 Data Supporting Information. (XLSX)

## Abbreviations:

RMSE: root mean square error
MAE: mean absolute error
MAPE: mean absolute percentage error
RNNs: recurrent neural networks
LSTMs: long short-term memory networks
TCN: temporal convolutional network
XGBoost: eXtreme Gradient Boosting
RF: random forests
HMM: hidden Markov models
MA: moving average
AR: auto regressive
SVM: support vector machines
CNN: convolutional neural networks
AutoML: automated model design
ARIMA: autoregressive integrated moving average
